# Risk assessment of coal mine water inrush based on *PCA-DBN*

**DOI:** 10.1038/s41598-022-05473-8

**Published:** 2022-01-25

**Authors:** Ye Zhang, Shoufeng Tang, Ke Shi

**Affiliations:** School of Information and Electrical Engineering, China University of Mining and Technology, Xuzhou, 221000 Jiangsu China

**Keywords:** Environmental sciences, Hydrology, Natural hazards

## Abstract

To provide an effective risk assessment of water inrush for coal mine safety production, a BP neural network prediction method for water inrush based on principal component analysis and deep confidence network optimization was proposed. Because deep belief network (DBN) is disadvantaged by a long training time when establishing a high-dimensional data classification model, the principal component analysis (PCA) method is used to reduce the dimensionality of many factors affecting the water inrush of the coal seam floor, thus reducing the number of variables of the research object, redundancy and the difficulty of feature extraction and shortening the training time of the model. Then, a DBN network was used to extract secondary features from the processed nonlinear data, and a more abstract high-level representation was formed by combining low-level features to find the expression of the nonlinear relationship between the characteristics of water in bursts. Finally, a prediction model was established to predict the water inrush in coal mines. The superiority of this method was verified by comparing the prediction of the actual working face with the actual situation in typical mining areas of North China. The prediction accuracy of coal mine water inrush obtained by this algorithm is 94%, while the prediction accuracy of traditional BP algorithm is 70%, and the prediction accuracy of SVM algorithm is 88%.

## Introduction

Deep mining of the Carboniferous Taiyuan Formation system is carried out in most North China-type coal mines. Because of its proximity to Ordovician limestone, water inrush accidents often occur. Therefore, the prediction of coal mine water inrush is a necessary part of coal mine safety production. The research and development of coal mine water inrush prediction are based on research on the mechanism of coal seam water inrush. Through this research on the mechanism of water inrush, many scholars in China and abroad have determined an index system for water inrush and defined the related influencing factors of water inrush. Regression analysis^1–4^, classification technology^5^, geographic information systems^6^, support vector machines^7–10^, neural networks^11^, extreme learning machine^12,13^ and other data analysis algorithms are applied to the prediction of coal mine water inrush, and the probability of water inrush accidents is analysed and evaluated, which provides data support for coal mine safety production. The law of influencing factors of coal mine water inrush accident is discrete and nonlinear. Multivariate discriminant analysis is a linear discriminant analysis model, such as Fisher model, and the accuracy rate may be very low when learning samples are inappropriate. BP neural and SVM have problems of slow convergence speed and local minimum point. When the latter is solving m-order matrix, large scale storage and calculation will consume machine memory and calculation time.

This paper proposes a coal mine water inrush prediction method based on the combination of PCA and DBN. PCA can effectively reduce the complexity of deep learning neural network diagnosis model, while DBN neural network not only has strong learning ability, but also has strong data feature extraction and feature transformation ability. This method has the advantages of strong anti-interference ability and high accuracy of prediction.

## Analysis of the influencing factors of water inrush

The occurrence of water inrush accidents in coal mines is the result of the joint action of many influencing factors. The interactions between the influencing factors that contribute to the water inrush accidents form a nonlinear system, which cannot be accurately expressed by the classic mathematical model. In China, the study of water inrush law began in the 1960s. The water inrush coefficient method was proposed by the Ministry of Coal, and the empirical formula of the water inrush coefficient was established^14^. Professor Jing Zigang of Shandong University of Science and Technology proposed the theory of "the next three belts"^15^. Dr. Liu Tianquan and Zhang Jincai of the General Institute of Coal Mine proposed the "two-zone" model, which considered that floor rock masses were composed of mining-induced water-conducting fissure zones and floor water-isolating zones^16^. In the twenty-first century, Professor Shi Longqing of Shandong University of Science and Technology put forward the theory of the "Lower Four Belts" based on the theory of "Lower Three. Belts"^17^. The Institute of Geology, Chinese Academy of Sciences, put forward the theory of a "strong seepage channel" in the 1990s, which believes that the presence of a water inrush channel is the key to the occurrence of water inrush^2^. Qian Minggao, an academician at the China University of Mining and Technology, proposed the KS theory of key strata of stope floor rock according to the layered structure characteristics of floor rock^18^. But most of these prediction methods for coal mine water inrush are limited to the evaluation of a key control factor, which is based on the calculation method of geological theory. The nonlinear dynamic characteristics of the occurrence process of water inrush and a va Main controlling factors of water inrush from coal seam floor.

5 major factors influence coal seam water inducing: confined aquifer, coalfield geological structure, Water barrier condition, aquifer performance and mine pressure failure development zone.

### Aquifer conditions

An aquifer conditions provides water and power for water inrush. The main influencing factors of a confined aquifer are aquifer water pressure, working face distance and aquifer thickness.

### Coal seam condition

The dip Angle of coal seam is the main factor affecting the depth of mining failure. The decrease of mining failure depth can effectively strengthen the thickness of waterproof layer. When the thickness of the coal seam is thick, it needs to be mined in layers, and each layer will destroy the integrity of the floor and reduce the waterproof performance.

### Structure condition

The geological structure provides water-inrush passages for water inrush, and main factors influencing water inrush accidents is faults. The main impacts of faults are, groundwater passage will be formed due to the stress damage caused by the fractured rock stratum and coal mining. And the mechanical strength of the fault zone rock is greatly reduced because of the tectonic stress.

### Water barrier condition

The coal seam aquifer is a water-repellent layer between the seam floor and the aquifer, which has an inhibitory effect on coal water inrush. The combination of lithology influenced the water barrier performance of the aquifer . Stratigraphic lithology indicate the mechanical strength of the rock layer or its ability to resist water pressure.

### Mining condition

The original pressure balance of the mine is destroyed by the mine excavation works, and the resulting changes in the geological and hydrological conditions of the coal seam will induce water-inrush accidents. The main influencing factors of confined water pressure are mining area, strike length and mining height. The Influencing factors of coal seam water inrush as shown in Table 1.

**Table 1:** Influencing factors of coal seam water inrush.

Aquifer conditions	Aquifer water pressure
	Working face distance
	Aquifer thickness
Water barrier condition	Thickness of argillaceous rock
	Thickness of Sandy Rock
	Thickness of limestone section
Coal seam condition	Coal seam thickness
	Coal seam dip angle
Structure condition	Fault fall
Mining condition	Mining area
	Strike length
	Mining height

## The theory of methods

### PCA

PCA is a dimension reduction algorithm. The principle is that the use of multiple indicators through linear transformation converts the comprehensive indicators of several unrelated indicators to each other, and according to certain rules to classify the integration of the comprehensive index, never reduces the dimension of the original data, extracts the main information in the original data, and minimizes the information loss in the process of the dimension reduction algorithm.

There is information overlap among the variables influencing the occurrence of water inrush, which will increase the cost and time of the classification prediction algorithm and reduce the success rate of its prediction. PCA is used to carry out dimensionality reduction processing on the original feature data, eliminate redundant information within the acceptable loss range, save the key evaluation index factors, and realize the dimensionality reduction of the evaluation index^19^.

### DBN

DBN is composed of a stack of several *Restricted Boltzmann machine (RBM)* and a classification or regression layer at the top. Through forward learning combined with the reverse fine-tuning mechanism of gradient descent, more accurate model training accuracy can be achieved.

### RBM

RBM is a probabilistic abrupt model that can be explained by a stochastic neural network. In the classic RBM structure, neurons located in the same layer have no correlation with each other. This structure is developed on the basis of a Boltzmann machine (BM), which solves the shortcoming of the unacceptably slow training speed of traditional RM and improves the training speed of the network^20^.

**Figure 1:**
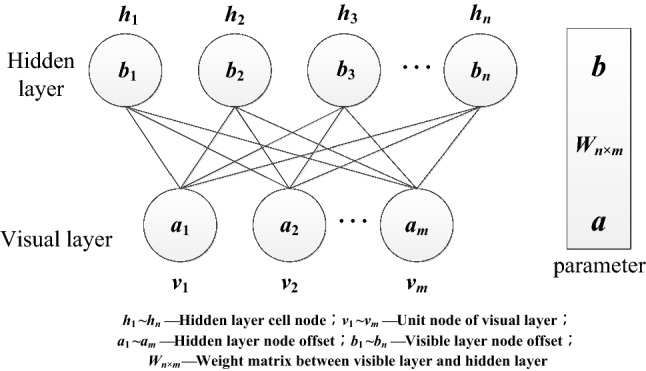
RBM structure diagram.

RBM is composed of two layers of neurons as shown in Fig. 1. There is undirected full connection between different neurons, and there is no connection between neurons in the same layer. Data are input by the visual layer and output by the hidden layer after training by neurons and weight matrix.

With given a cell node (*v*, *h*), the energy function of RBM is

1 $$\begin{aligned} E(v,h) &= - {a^T}h - {b^T}v - {v^T}{w_{n \times m}}h \\ &= - \sum {_i{b_i}{v_i} - } \sum {_j{a_j}{h_j} - \sum {_{i,j}{w_{ij}}{v_i}{h_j}} } \\ \end{aligned}$$

Based on the energy function, the following probability distribution under the condition *Θ* = (*w*_*n*×*m*_*, a, b*) can be obtained:

2 $${p_\Theta }(v,h) = \frac{{{e^{{E_\Theta }(v,h)}}}}{{\sum {_{v,h}{e^{{E_\Theta }(v,h)}}} }}$$

3 $${p_\Theta }(v) = \sum {_h} {p_\Theta }(v,h) = \frac{1}{Z}\sum {_h} {e^{{E_\Theta }(v,h)}}$$

4 $${p_\Theta }(h) = \sum {_v} {p_\Theta }(v,h) = \frac{1}{Z}\sum {_v} {e^{{E_\Theta }(v,h)}}$$

*Z* is the normalized coefficient.

The activation probabilities of h and v are obtained after the activation function sigmoid:

5 $$p({h_j} = 1\left| v \right.) = sigmoid({a_i} + \sum {_i} {v_i}{w_{ij}})$$

6 $$p({v_i} = 1\left| h \right.) = sigmoid({b_j} + \sum {_j} {h_j}{w_{ij}})$$

The core formula of the RBM algorithm is the activation formula of *h* and *v*. Data are input from the visual layer, and the characteristic index is mapped from the visual layer to the neurons of the hidden layer through Eq. (5). Then, the output value obtained is reconstructed to the visual layer *v* through Eq. (6), and the error between the reconstructed data in the original data domain is calculated. The weight parameters between the visible and hidden layers are adjusted by the error minimization rule so that the reconstructed data can represent the original input data to the maximum and achieve the goal of feature extraction. In fact, the goal of the training process of the RBM algorithm is to solve the Markov maximum likelihood estimation problem; that is, under the condition of fixed data input, the *P*_*Θ*_*(v)* value is maximized by adjusting the internal parameters of the RBM.

### DBN network structure

A DBN is composed of multiple stacked RBMs, which construct a typical DBN network model. Compared with the shallow neural network, this kind of stacked DBN structure has a deeper network level and better model generalization ability. Traditional neural networks rely on the selection of data features, while DBN can extract hidden features from input data by setting multiple hidden layers^21^.

**Figure 2:**
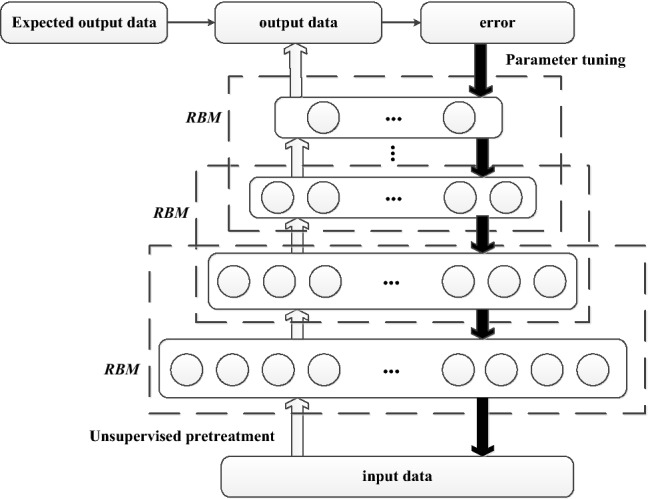
DBN model.

The DBN is composed of a cascading RBM and a back propagation algorithm adopted in the top layer as shown in Fig. 2. The algorithm training process is divided into two parts: pretraining and parameter fine-tuning. Pretraining means that the input data are trained layer by layer unsupervised by the bottom RBM, and the output of the previous layer will be used as the input data of the upper-layer RBM. This structure can effectively screen out the feature information. The parameter fine-tuning process involves overall tuning and supervised training. The error between the expected data in the output data domain is propagated back layer by layer to fine-tune the parameters of the entire network^22^. The original data is shown in Table 2.

**Table 2:** Original data.

Water pressure	Distance	Aquifer thickness	Argillaceous rock	Sandy Rock	Limestone section	Coal seam	Dip angle	Fault fall	Mining area	Strike length	Mining height
15	12.31	3.4	88.871	7.067	4.062	0	25	10	746	22	1.4
23.1	39.66	5.75	0	0	0	0	9.25	5	14,130	122	1.45
17.85	31.58	5.79	0	0	0	0	15	4	13,304	100	1.45
8.48	32	5.5	0	0	0	0	10	1	1000	20	1.7
21.6	30.53	5.99	67.9	12.643	13.528	5.929	13.5	1.6	346.5	15	1.55
17.29	43.45	5.39	0	0	0	0	10.5	1.5	7284	136	1.65
18.08	40	4.16	40.275	40.375	14.85	4.5	7.5	1.3	16,160	162	1.55
…	…	…	…	…	…	…	…	…	…	…	…
8.1	16.1	4.49	97.205	0	2.795	0	12	1.5	1040	31	1.5
24	45.11	6.17	0	0	0	0	10	11	21,857	258	1.45
25.12	41.11	5.297	34.374	62.659	1.095	1.873	7	5	972	63	1.4

### The DBN prediction model

The coal mine water inrush accident data presents non-linear, high-dimensional characteristics, and there are complex interrelationships among various water inrush accident-related factors. Most of the current prediction and evaluation methods cannot effectively extract a large number of hidden features in the data, resulting in a more partial water inrush accident model, which affects the prediction accuracy and cannot provide effective support for safely mining in coal mines. Therefore, there are two main aspects of model design ideas in this paper: converting high-dimensional influencing factors into low-dimensional, easy-to-train data and more complete extraction of features in the data^23^.

### The PCA data dimensionality reduction

The PCA algorithm is used to perform nonlinear dimensionality reduction on the main control factors of coal mine water inrush and to standardize the data proof of the coal mine's actual sampling. SPSS software is used to perform principal component analysis on the corresponding measured data. The selection criterion of principal components is that the cumulative variance contribution rate must exceed 80%. Since the cvcp value of the first to the sixth principal component is approximately 83%, these six components contain most of the information required for water inrush prediction, and thus, the first 6 components are used for floor water inrush evaluation. The contribution rate and cumulative contribution rate of principal components are shown in Table 3.

**Table 3:** Contribution rate and cumulative contribution rate of principal components.

Element	vcp (%)	cvcp (%)
1	32.38	32.38
2	15.67	48.05
3	13.52	61.57
4	10.01	71.58
5	6.02	77.6
6	5.82	83.4

### DBN model training

Use SMOTE algorithm to expand 100 sets of data into 300 sets of water inrush datasets, and the PCA is used to reduce dimensionality. The reduced-dimensional data is input into the DBN for pretraining. The pretraining first initializes the weight matrix between each layer, traverses the input vector and the hidden layer neuron nodes, and then outputs the neuron parameters after the first RBM is trained. As the input vector of the second RBM, it is finally passed layer-by-layer to the highest layer. According to the results of the pretraining output layer, the error between it and the expected output backpropagation from each output layer to the hidden layer updates the parameters of each layer^16^.

**Figure 3:**
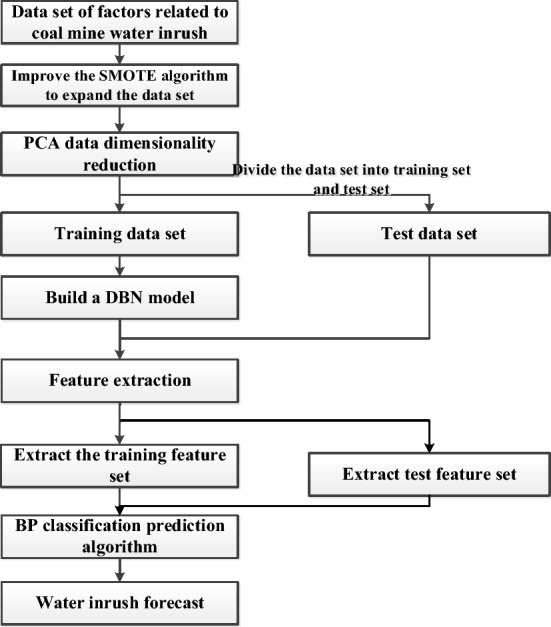
Algorithm flow chart.

The advantage of the model lies in the use of DBN abstraction to extract output data features and a neural network as the top-level unit of DBN to predict water inrush after extracting new features. The model prediction process is shown in Fig. 3.

A 4-layer RBM network is established, the number of input layer nodes is determined by the data dimension, and the number of hidden layer nodes is obtained by the ‘trial and error method.

The unlabeled training data is input into the DBN network, the RBM parameters are pretrained layer-by-layer, and we optimize the RBM parameters locally.

The labeled training data is input, the error back is propagated layer-by-layer, and the gradient descent method is used to update the weights of the DBN network until convergence.

All data is input into the network for feature learning, and the output reconstructed feature data is extracted.

The feature data is divided into test and training data, the labeled training data is input into the BP neural network for training, and the trained network is used to predict the test set. The predicted water inrush situation is compared with the actual situation and the prediction result is evaluated.

### Algorithm verification

The proposed method is used to test the water inrush data of the measured working face in a typical mining area in North China, and the water inrush situation is predicted and compared by DBN, SVM, BP and other classic algorithms. The three types of modeling use the same number of samples, which are all data after oversampling. After the original data is processed by the PCA, the dimensionality of the feature values is reduced to 6. The algorithm is written in Python.

**Figure 4:**
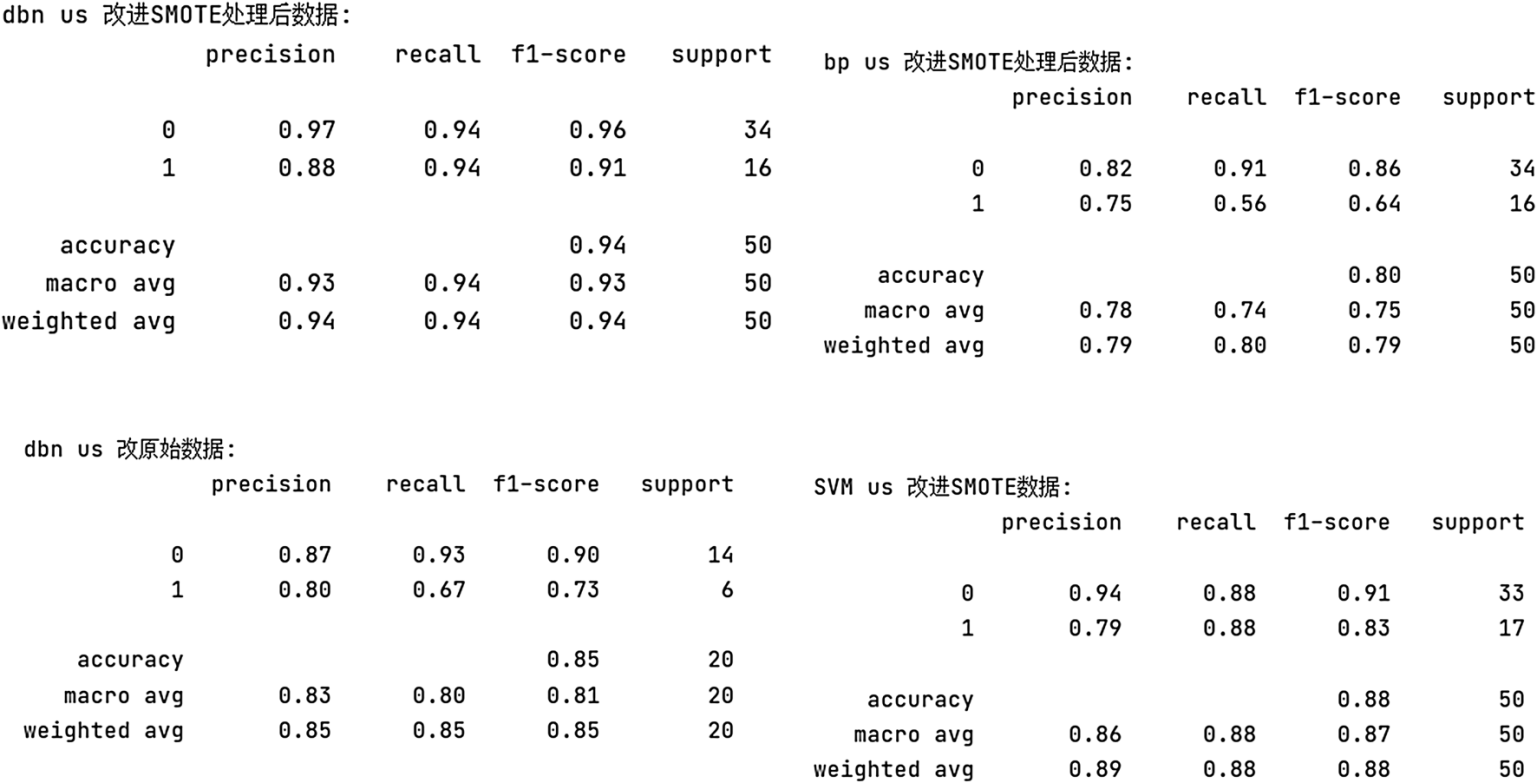
Ablation experiment.

**Figure 5:**
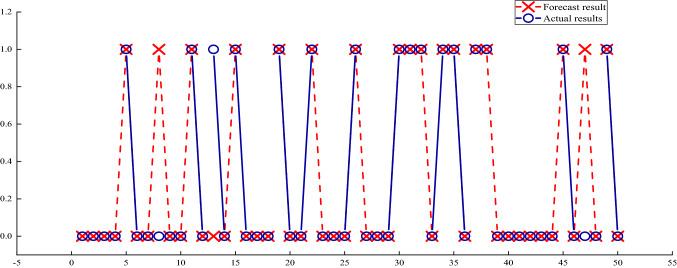
The prediction results.

The data in Table 4 is entered into the DBN model, and the results are shown in Table 5, Figs. 4 and 5. There are three incorrect predictions, which means that the correct rate is 94%. The reason for the incorrect prediction sample may be the result of an insufficient sample size and missing features in dimensionality reduction. In the training process, better dimensionality reduction methods can improve the accuracy of the algorithm. The correct rate of the BP neural network using oversampling data is 80%, the correct rate of the water burst coefficient method is 60%, and the SVM algorithm using the SMOTE oversampling data is 88%, and the accuracy rate of the DBN algorithm trained with the unexpanded data training set is 85%. It can be seen from Table 6 that the accuracy rates of the water inrush risk prediction models proposed in this paper are better than the rates of these method.

**Table 4:** Part of the data after PCA dimensionality reduction.

No	s1	s2	s3	s4	s5	s6	Result
1	0.117647	0.435	0	0.23	0	0.4307	0
2	0.647059	0.675	0	0	0	0.779434	1
3	0.235294	0.495	0.590164	0.17	0	0.38152	0
4	0.352941	0.48	0	0	0	0.353204	0
5	0.117647	0.555	0.491803	0	0	0.390462	0
6	0.705882	0.53	0.655738	0	0	0.351714	0
7	0.176471	0.435	0.491803	0.18	0	0.324888	0
8	0.294118	0.615	0.606557	0.4	0.091017	0.508197	0
9	0.117647	0.45	0.491803	0	0	0.350224	0
10	0.470588	0.545	0.622951	0.39	0.134752	0.415797	1
11	0.176471	0.56	0.606557	0.3	0	0.470939	1
12	0.058824	0.665	0.836066	0.28	0.165485	0.488823	1
…	…	…	…	…	…	…	…
247	0.411765	0.905	0.688525	0.21	0.22695	0.535022	1
248	0.352941	0.97	0.639344	0	0	0.350224	1
249	0	0.755	0.737705	0.46	0	0.627422	0
250	0.529412	0.725	0.655738	0.46	0.153664	0.564829	1

**Table 5:** Comparison of model results and actual results.

Water inrush instance number	Forecast result	Actual results	Whether it meets the measured results
251	0	0	Y
252	0	0	Y
253	0	0	Y
254	0	0	Y
255	1	1	Y
256	0	0	Y
257	0	0	Y
258	0	1	N
259	0	0	Y
260	0	0	Y
261	1	1	Y
262	0	0	Y
263	1	0	N
264	0	0	Y
…	…	…	…
297	0	1	N
298	0	0	Y
299	1	1	Y
300	0	0	Y

**Table 6:** Comparison of prediction results of different models.

Methods	Time/s		Accuracy rate/%	
	Training set	Test set	Training set	Test set
BP	0.5822	0.0154	84.98	80
SVM	0.0057	0.0032	85.23	88
DBN	0.0062	0.0031	86	85
PCA-ELM	0.0024	000,012	85.51	90.80
LLE-SVM	0.0034	0.0016	84.35	90
PCA-DBN	0.0022	0.0009	87.17	94

The model proposed in this paper can be directly applied to the prediction of water inrush from coal fields in North China. The prediction results show that DBN can effectively extract features. DBN has good performance for nonlinear and interrelated data, such as water inrush influencing factors. The preprocessing function can effectively improve the prediction effect of the BP neural network. In summary, the DBN prediction model based on PCA has a good predictive effect on water inrush data. It can also make a more accurate water inrush risk assessment for coal mine safety production.

## Conclusion

There are many risk factors affecting coal floor water inbursts, and some data are redundant. Principal component analysis reduces the data dimension without damaging the integrity of the data and saves the cost of the training algorithm. By training relative to the original features of PCA and BP, the PCA-DBN model is more effective for extracting the characteristics of water inrush that influence the original data, improving the training accuracy and generalizing the performance of the model. As a result, the PCA-DBN model can eliminate the defects of traditional algorithms for feature selection, extract implicit characteristics in complex hydrogeological information, and effectively filter the missing and noise data to establish a more reliable evaluation model for water inrush accidents. The case analysis shows that the predicted value of the model is consistent with the actual situation of water inrush in coal mines, and the following conclusions are drawn:

1. The multidimensional redundant input data will complicate the structure of the DBN. PCA is used to reduce the dimensionality of the data, extract the nonlinear features of the high-dimensional data, and input them into the deep confidence network, which can simplify the network structure and improve the accuracy of the model.
2. Compared with the traditional BP network, the PCA-BP network model and the water inburst coefficient method, the PCA-DBN model proposed in this paper has the highest prediction accuracy. In subsequent research, the network model can be optimized from the structure of the DBN network itself, and other algorithms can be integrated to further improve the model’s accuracy^24–26^.
